# Prediction of Rockfill Materials’ Shear Strength Using Various Kernel Function-Based Regression Models—A Comparative Perspective

**DOI:** 10.3390/ma15051739

**Published:** 2022-02-25

**Authors:** Mahmood Ahmad, Ramez A. Al-Mansob, Irfan Jamil, Mohammad A. Al-Zubi, Mohanad Muayad Sabri Sabri, Arnold C. Alguno

**Affiliations:** 1 Department of Civil Engineering, Faculty of Engineering, International Islamic University Malaysia, Jalan Gombak 50728, Selangor, Malaysia; ahmadm@iium.edu.my; 2 Department of Civil Engineering, University of Engineering and Technology Peshawar (Bannu Campus), Bannu 28100, Pakistan; 3 Department of Civil Engineering, University of Engineering and Technology Peshawar, Peshawar 25000, Pakistan; irfanuop@hotmail.com; 4 Department of Mechanical Engineering, Hijjawai Faculty for Engineering, Yarmouk University, Irbid 21163, Jordan; mohammad.alzubi@yu.edu.jo; 5 Peter the Great St. Petersburg Polytechnic University, 195251 Saint Petersburg, Russia; mohanad.m.sabri@gmail.com; 6 Department of Physics, Mindanao State University-Iligan Institute of Technology, Iligan City 9200, Philippines; arnold.alguno@g.msuiit.edu.ph

**Keywords:** shear strength, rockfill materials, Gaussian functions, polynomial kernel, radial basis function, Pearson universal kernel

## Abstract

The mechanical behavior of the rockfill materials (RFMs) used in a dam’s shell must be evaluated for the safe and cost-effective design of embankment dams. However, the characterization of RFMs with specific reference to shear strength is challenging and costly, as the materials may contain particles larger than 500 mm in diameter. This study explores the potential of various kernel function-based Gaussian process regression (GPR) models to predict the shear strength of RFMs. A total of 165 datasets compiled from the literature were selected to train and test the proposed models. Comparing the developed models based on the GPR method shows that the superlative model was the Pearson universal kernel (PUK) model with an R-squared (R^2^) of 0.9806, a correlation coefficient (*r*) of 0.9903, a mean absolute error (MAE) of 0.0646 MPa, a root mean square error (RMSE) of 0.0965 MPa, a relative absolute error (RAE) of 13.0776%, and a root relative squared error (RRSE) of 14.6311% in the training phase, while it performed equally well in the testing phase, with R^2^ = 0.9455, *r* = 0.9724, MAE = 0.1048 MPa, RMSE = 0.1443 MPa, RAE = 21.8554%, and RRSE = 23.6865%. The prediction results of the GPR-PUK model are found to be more accurate and are in good agreement with the actual shear strength of RFMs, thus verifying the feasibility and effectiveness of the model.

## 1. Introduction

In civil engineering projects, such as rockfill dams, slopes, and embankments, rockfill materials (RFMs) are often used as filling materials. RFMs consist of coarse gravels, cobbles, and boulders mined from rock quarries or riverbeds. Quarried materials are angular to sub-angular, whereas riverbed materials are rounded to sub-rounded. Mineral composition, particle size, shape, gradation, individual particle strength, void content, relative density, and surface roughness of the particles all influence the behavior of the RFMs utilized in the construction of rockfill dams. Several studies in geotechnical engineering have been carried out, such as that examining the contact between the soils and concrete used in earth and rockfill dams [1]. Inverse analysis provides an means to better understand dam behavior [2], and offers measures of surface roughness, apparent porosity, apparent density, water absorption, and uniaxial compression strength (UCS), as well as an understanding of the influence of the heating rate and cooling process of gneiss stone [3].

RFMs are the preferred materials for the construction of high-embankment dams in seismically vulnerable areas because they provide structural flexibility and considerably reduce the issues caused by porewater pressure during and after construction. The mechanical behavior of the RFMs utilized in the dam’s shell must be evaluated. However, because the materials may contain pieces larger than 500 mm in diameter, the characterization of RFMs with special reference to shear strength and deformation is challenging and expensive.

A number of studies have been carried on RFM behavior. Marsal [4], Mirachi et al. [5], Venkatachalam [6], Gupta [7], Abbas [8], and Honkanadavar and Sharma [9] conducted laboratory experiments on different RFMs and found that the behavior of stress–strain is nonlinear, inelastic, and dependent on the stress level. They also found that as the maximum particle size for riverbed RFM increases, so does the angle of internal friction, but quarry RFM shows the reverse trend. Frossard et al. [10] developed a logical method for evaluating RFMs’ shear strength based on size effects. To associate the shear strength parameter to some riverbed RFM index features, Honkanadavar and Gupta [11] developed a power law. Due to the difficulty of performing large-scale strength tests and characterizing the mechanical behavior of RFMs, different approaches for predicting their behavior have been developed. In laboratory experiments, RFM with a large particle size (maximum particle size of 1200 mm) was determined to be incompatible [11]. Large-scale shear tests are time-consuming and difficult, and estimating the nonlinear shear strength function without an analytical method is complicated.

In the recent past, machine learning (ML) algorithms have achieved notable successes at efficiently solving real-world problems in different sectors, including civil and environmental engineering [12], geotechnical engineering [13,14,15,16,17,18], and other fields of science [19,20,21,22,23,24,25,26]. The artificial neural network (ANN) approach was found to be more efficient in predicting the shear strength of RFMs [27]. Zhou et al. [28] has recently shown that cubist and random forest regression algorithms are better at predicting RFM shear strength results than ANN and traditional regression models. To predict RFMs’ shear strength, Ahmad et al. [29] used support vector machine, random forest, adaptive boosting, and k-nearest neighbor algorithms. This field, on the other hand, is still being researched and further explored.

No prior study was found to simultaneously evaluate the effectiveness of a range of kernel function-based Gaussian process regression (GPR) models. The goal of this research is to evaluate and compare the efficacy of various kernel function-based GPR models based on the results of reliable experimental tests of shear strength prediction modeling for rock materials. The aims of this research include the following:

- To examine the capability of various kernel function-based GPR computing techniques, namely, radial basis function kernel, polynomial kernel, and Pearson universal kernel, in the area of predicting the shear strength of RFMs;
- To undertake a comparative study of the shear strength prediction of rockfill materials and select the best outcomes provided by the developed GPR models based on the performance metrics;
- To conduct sensitivity analyses to determine the effect of each input parameter on the RFMs’ shear strength.

The remainder of the paper is organized as follows. In Section 2, the details of three different types of kernel function-based GPR computing techniques for predicting the shear strength of rockfill materials are presented. In Section 3, the details of the data catalog and correlation analysis are presented. Performance evaluation measures are presented in Section 4. Section 5 reports the developed models’ results. Based on the observations and results of these models, Section 5 draws conclusions and future research directions.

## 2. Gaussian Process Regression

**Figure 1:**
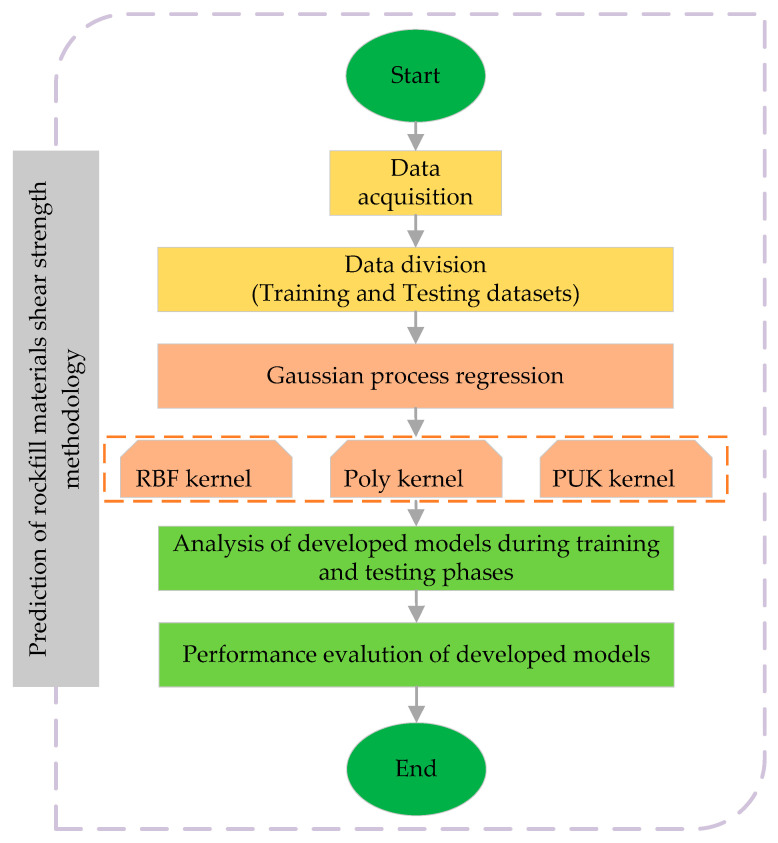
Flowchart of the proposed methodology.

Gaussian process regression (GPR) is a suitable and recently described method that has been used in a variety of machine learning applications [30]. The GPR model’s probabilistic solution leads to the identification of general kernel regression problems. The applied regressor’s training process can be categorized as Bayesian, and the model relations are assumed to follow a Gaussian distribution to encode the previous output function information [31]. The Gaussian process is defined by a set of variables, each of which has a joint Gaussian allocation [32]. Figure 1 illustrates the scheme of development of the selected methodology.

### 2.1. Radial Basis Function Kernel

The RBF kernel is a typical kernel function used in several machine learning algorithms for kernel learning. It is widely used in vector machinery classification. RBF kernels are typically a decent first option. Instead of the usual kernel, this kernel transfers samples to higher-dimensional areas, allowing a nonlinear relationship between class labels and attributes to be dealt with [33]. Furthermore, with the linear kernel and normalized poly kernel, RBF is a unique situation because the linear kernel with a penalty parameter C operates with the same parameters as the RBF kernel.

In comparison, kernel values can be used to calculate $\gamma X_{i}^{T}+r>1$ for polynomial kernels up to infinity as long as the grade is high [34]. Furthermore, under such parameters, the sigmoid kernel is not true, i.e., it is not an internal two-vector product. The RBF kernel is not appropriate in several cases. The linear kernel can be employed in particular when the number of functions is quite large [35].

### 2.2. Polynomial Kernel

The polynomial kernel (poly kernel) is a kernel function often used with support-vector machines (SVMs) and other kernel-coded models that relate to parallel vectors in the machine learning language. The polynomial kernel appears to achieve similarity not only on the input samples’ stated functions, but also in their combinations. In the context of regression analysis, such groupings were categorized as interaction features. These groupings have been identified as interaction features in the context of regression analysis. The enclosed polynomial kernel space is the same as a polynomial regression, but it is an educated sum of parameters that does not include a combined blow-up. The functionality links to logical function connections if the function data input are binary [36]. The polynomial kernel for degree polynomials is well-defined as

(1) $$K\left(x,Y\right)={\left(x^{T},y+C\right)}^{d}$$

where x and y are vectors in the input space, i.e., feature vectors found from training or trial samples, and C≥0 is an unconstrained parameter that trades off higher-order vs. lower-order polynomial definitions. When C=0, the kernel is said to be homogeneous.

### 2.3. Pearson Universal Kernel

The Pearson universal kernel (PUK) is a machine learning programming tool that aids in the comprehensive interpretation and understanding of various data types. The Pearson VII function is considered to have a general form for curve suitably, and is assumed by

(2) $$f\left(x\right)={H/\left[1+2\left(x-x_{0}\right)\sqrt{2{\left({}^{\left(\frac{1}{\omega }-1\right)/\delta }\right)}^{2}}\right]}^{\omega }$$

where H is the top tallness at the middle $x_{0}$ of the peak, and x represents the self-determining variable. The parameters σ and ω regulate the half-width and the following factor of the peak. A function ω, on the other hand, belongs to the class of effective kernel functions. The kernel matrix might be symbolic and positively semi-definite; to show that PUK is certainly resolving these situations, Uestuen [37] redrafted Equation (3) into a function of both vectors:

(3) $$K\left(x_{i},x_{j}\right)=\frac{1}{{\left[1+((2\sqrt{{\left|x_{i}-x_{j}\right|}^{2}\sqrt{2^{\left(\frac{1}{\omega }\right)}-1)}/\sigma )}\right]}^{2\omega }}$$

## 3. Data Catalog and Correlation Analysis

The dataset collected from Kaunda [27] was separated into training (80 percent of total data) and testing (20 percent of the remaining data) datasets for this investigation. The database has been presented in Kaunda [27] in detail. Table 1 summarizes the 165-sample dataset, which includes numerous shear strength of RFM tests, and the input and output variables’ minimum (min), maximum (max), mean, and standard deviation (SD). As can be seen in the table, the database includes input parameters, i.e., particle material size (or sieve) gradation, fineness modulus, gradation modulus, material hardness, relative density, and confining (normal) stress, and one output parameter, i.e., shear strength.

To choose the most resilient representation, a statistical study of input and output variables of the training and testing data was performed (see Table 2). It was accomplished through the use of a trial-and-error strategy. Previous studies show that the shear strength (τ) of RFM is a function of *D*_10_, *D*_30_, *D*_60_, and *D*_90_, which correspond to the 10%, 30%, 60%, and 90% passing sieve sizes, while *UCS_min_* and *UCS_max_* (MPa) indicate the minimum and maximum uniaxial compressive strengths (MPa), the *FM* and *GM* parameters describe fineness modulus and gradation modulus, respectively, *γ* is the dry unit weight (kN/m^3^), *σ_n_* is the normal stress (MPa), and *R* shows the International Society of Rock Mechanics (ISRM) hardness rating [27,29]. As a result, the current study’s GPR models are constructed using these input variables.

Understanding the relationship between each input and result can definitely facilitate the development of a proper prediction model. Among the numerous correlation coefficients described thus far, the correlation coefficient technique has proven to be more common. As stated in Equation (4), the correlation coefficient is equal to the product of the covariance of two parameters divided by their standard deviation. $\rho_{m,n}\approx 1$ represents the high degree of interdependence between two variables, while $\rho_{m,n}\approx 0$ stands for a linear relationship between two variables m and n that are independent of one another. The Pearson correlation coefficients for the variable inputs and the target output are reported in Table 3.

(4) $$\rho \left(m,n\right)=\frac{\mathrm{cov}\left(m,n\right)}{\sigma_{m}\sigma_{n}}$$

Obviously, the values provided in Table 3 reveal that *σ_n_* and *D*_90_ have the most significant influence on the *τ*, while *FM*, *C_u_*, and *UCS_max_* affect the output non-considerably.

## 4. Performance Evaluation Measures

The coefficient of determination (R^2^), Pearson’s correlation coefficient (*r*), mean absolute error (MAE), root mean square error (RMSE), relative absolute error (RAE), and root relative squared error (RRSE) are used to evaluate the data-driven modeling in this study. These parameters can be calculated as follows:

(5) $$R^{2}=1-\frac{\sum_{i=1}^{n}{\left(y_{i_{p}}-y_{i_{o}}\right)}^{2}}{\sum_{i=1}^{n}{\left(y_{i_{o}}-{\bar{y}}_{o}\right)}^{2}}$$

(6) $$r=\frac{\sum_{i=1}^{n}\left[\left(y_{i_{o}}-{\bar{y}}_{p}\right)\left(y_{i_{o}}-{\bar{y}}_{p}\right)\right]}{\sqrt{\sum_{i=1}^{n}{\left(y_{i_{o}}-{\bar{y}}_{p}\right)}^{2}}\sqrt{\sum_{i=1}^{n}{\left(y_{i_{o}}-{\bar{y}}_{p}\right)}^{2}}}$$

(7) $$\mathrm{MAE}=\frac{1}{N}\sum_{i=1}^{n}\left|y_{i_{o}}-y_{i_{p}}\right|$$

(8) $$\mathrm{RMSE}=\sqrt{\frac{1}{N}\sum_{i=1}^{n}{\left(y_{i_{o}}-y_{i_{p}}\right)}^{2}}$$

(9) $$\mathrm{RAE}=\frac{\sum_{i=1}^{n}\left|y_{i_{p}}-y_{i_{o}}\right|}{\sum_{i=1}^{n}\left|y_{i_{o}}-{\bar{y}}_{o}\right|}$$

(10) $$\mathrm{RRSE}=\frac{\sqrt{\sum_{i=1}^{n}{\left(y_{i_{p}}-y_{i_{o}}\right)}^{2}}}{\sqrt{\sum_{i=1}^{n}{\left(y_{i_{o}}-{\bar{y}}_{o}\right)}^{2}}}$$

where $y_{i_{o}}$ and $y_{i_{p}}$ represents the actual measurement and predicted shear strength of RFM, respectively, ${\bar{y}}_{o}$ is the average of the reference samples’ values, and n is the defined amount of data.

The degree of collinearity between predicted and measured data is described by the coefficient of determination (R^2^) and correlation coefficient (*r*). The correlation coefficient, which varies from 1 to −1, is a measure of the degree to which observed and predicted data are linearly related. There is no linear relationship if *r* = 0. A perfect positive or negative linear relationship arises if *r* = 1 or −1. Similarly, R^2^ denotes the percentage of variance in the measured data that the model can explain. R^2^ spans from 0 to 1, with higher values suggesting less error variation, and values above 0.5 are usually regarded as acceptable [38,39]. The MAE represents the average value of the predicted and actual values. When the MAE is close to 0, the adjustment has a better effect, implying that the prediction model more accurately describes the set of training data [40]. As a single measure of predictive power, the RMSE is the average magnitudes of the errors in predictions for all observations. The RMSE is greater than or equal to 0, with 0 indicating a statistically perfect fit for the observed data. The relative absolute error (RAE) is the difference between expected and actual values that is calculated by dividing the mean difference by the arithmetic mean. It ranges from 0 to infinite, with being 0 the best value. The RRSE criteria measure the model’s percentage error, which ranges from 0 to 100. Consequently, the better the model, the lower the values of these criteria are. Furthermore, visual inspections, such as scatter plots, were used to compare the performance of the developed models.

## 5. Results and Discussion

### 5.1. Comparative Performance

In this paper, various kernel function-based Gaussian process regression techniques were implemented using Waikato environment for knowledge analysis (WEKA). WEKA is an open-source software which consists of a collection of machine learning algorithms for data mining tasks. Most machine learning algorithms have hyperparameters that must be tuned. The critical hyperparameters in the GPR-RBF, GPR-Poly, and GPR-PUK models are tuned in this study as shown in Table 4. The values for the models’ tuning parameters were chosen first, and then varied in the trials until the best fitness measures in Table 4 were obtained.

Table 5 shows the R^2^, *r*, MAE, RMSE, RAE, and RRSE values for shear strength estimation after hyperparameter tuning for the training and testing phases, respectively. At a glance, for both tables, GPR-PUK is the top-ranked model. For the training results, based on the R^2^ (0.9224, 0.9430, and 0.9806), *r* (0.9604, 0.9711, and 0.9903), MAE (0.1565, 0.1015, and 0.0646), RMSE (0.2219, 0.1605, and 0.0965), RAE (31.7002%, 20.5572%, and 13.0776%), and RRSE (31.7002%, 20.5572%, and 13.0776%), respectively, for GPR-RBF, GPR-Poly, and GPR-PUK models, the GPR-PUK outputs are verified to be the most compatible with actual RFM shear strength values. Following that, GPR-Poly demonstrated a high level of accuracy. Furthermore, the findings obtained for the testing dataset demonstrate GPR-PUK’s superior performance. Considering the respective values of R^2^ (0.9334, 0.9411, and 0.9455), *r* (0.9661, 0.9701, and 0.9724), MAE (0.1395, 0.1092, and 0.1048), RMSE (0.1781, 0.1508, and 0.1443), RAE (29.0963%, 22.783%, and 21.8554%), and RRSE (29.2377%, 24.7641%, and 23.6865%), respectively, for the GPR-RBF, GPR-Poly, and GPR-PUK models, GPR-Poly is the second most precise model, and GPR-PUK has outperformed both GPR-RBF and GPR-Poly. In comparison with the ANN model (R^2^ = 0.9386) and linear regression method (R^2^ = 0.836) reported by Kaunda [27] and Andjelkovic et al. [41], respectively, for the test data, the proposed GPR-PUK (R^2^ = 0.9455) has better prediction capacity. In general, the generalization and reliability of the GPR-PUK perform well, and larger datasets can yield better prediction results.

**Figure 2:**
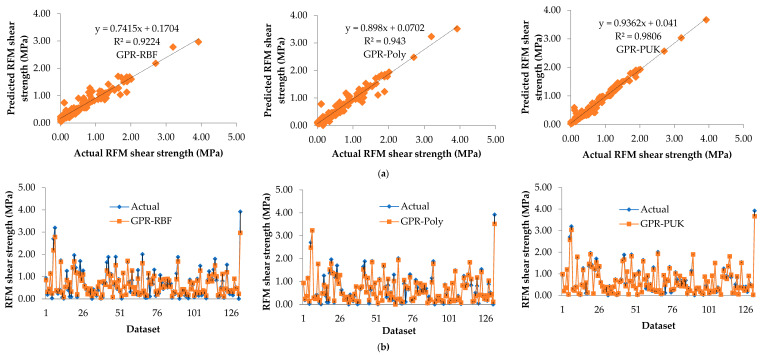
Comparison of the results in the training dataset of the various kernel function-based Gaussian process regression (GPR) models (**a**) measured vs. predicted RFM shear strength, (**b**) showing the accuracy of the models in predicting RFM shear strength.

**Figure 3:**
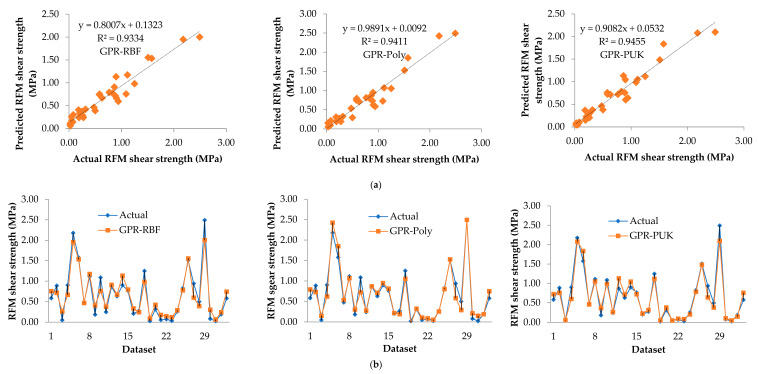
Comparison of the results in the testing dataset of the various kernel function-based Gaussian process regression (GPR) models (**a**) measured vs. predicted RFM shear strength, (**b**) showing the accuracy of the models in predicting RFM shear strength.

In addition, for the GPR-RBF, GPR-Poly, and GPR-PUK models, the graphical correlation between measured (on the horizontal axis) and predicted (on the vertical axis) shear strength is presented in Figure 2a for the training dataset and Figure 3a for the testing dataset, respectively. The trend line for GPR-PUK has been drawn by comparing the observed regression in Figure 2a and Figure 3a, and the GPR-PUK findings have the maximum inclination to the line of y=x (i.e., R^2^ = 0.9806).

The accuracy of all the developed models i.e., GPR-RBF, GPR-Poly, and GPR-PUK in predicting RFM shear strength is illustrated in Figure 2b for the training dataset and Figure 3b for the testing dataset, respectively. As seen in this graph, the closer one moves to the y axis (i.e., the lower the error), the higher the accuracy in both the training and testing datasets. Here, the GPR-PUK model has presented the most reliable prediction. This is evident by the higher aggregation of the results around the y axis (y=0), except for a few noise points. In comparison to the other models, i.e., GPR-RBF and GPR-Poly, the comparison findings are sufficiently consistent, which is adequate for the proposed GPR-PUK model to predict RFM shear strength values.

### 5.2. Rank Analysis

Rank analysis assigns score values to statistical parameters, using their ideal values as a benchmark, based on the number of models utilized. The model with the best performance receives the highest score, and vice versa. Table 6 shows how the total efficiency ranking of the developed models is interpreted using a rank analysis (i.e., the summation of the ranks of R^2^, *r*, MAE, RMSE, RAE, and RRSE into a single ranking score for the training and testing datasets). Based on the obtained total ranking scores of 12, 24, and 36 (respectively for GPR-RBF, GPR-Poly, and GPR-PUK), the superiority of GPR-PUK can be concluded (i.e., it has the most significant total rank). The GPR-Poly model has commonly been labeled as the second most accurate model. The most significant point about Table 6 is that the GPR-RBF, GPR-Poly, and GPR-PUK predictive models achieved the same rank for each measure. The GPR-PUK and GPR-RBF, for instance, had the highest (3) and lowest (1) level of accuracy, based on the R^2^, *r*, MAE, RMSE, RAE, and RRSE indices simultaneously.

### 5.3. Sensitivity Analysis

**Figure 4:**
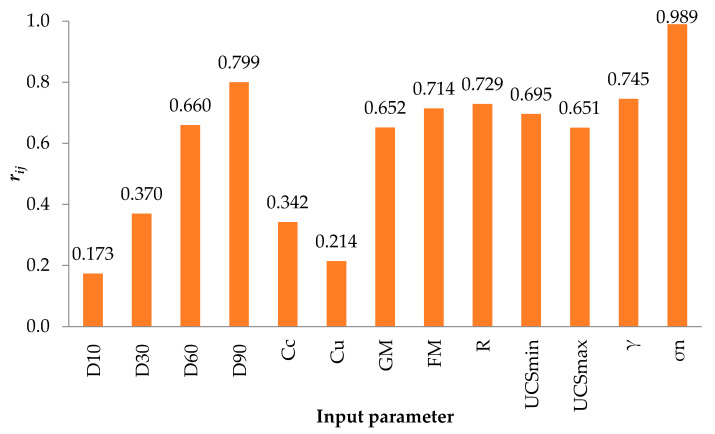
Sensitivity analysis of the input parameter.

Yang and Zang’s [42] sensitivity analysis was used to analyze the developed models’ ability to analyze the impact of input variables on the shear strength of rockfill material. This method has been used in several research studies [43,44,45,46], and is as follows,

(11) $$r_{ij}=\frac{\sum_{m=1}^{n}\left(y_{im}\times y_{om}\right)}{\sqrt{\sum_{m=1}^{n}y_{im}{}^{2}\sum_{m=1}^{n}y_{om}{}^{2}}}$$

where n is the number of data values, and $y_{im}$ and $y_{om}$ are the input and output parameters. For each input parameter, the $r_{ij}$ value varied from zero to one, with the highest values indicating the most efficient output parameter (which was τ in this study). To estimate the relationship between input and output variables, the value of *r_ij_* must be close to 1. Figure 4 shows the degree of importance of the input variables based on the experimental actual and predicted values of the shear strength. As it can be seen, the importance of different parameters can be displayed as *σ_n_* > *D*_90_ > *γ* > *R* > *FM* > *UCS_min_*> *D*_60_ > *GM* > *UCS_max_* > *D*_30_ > *C_c_* > *C_u_* > *D*_10_. In other words, the *σ_n_* is the most important parameter, and the D_10_ is the least important parameter for predicting the shear strength of the RFMs. Furthermore, Table 3 shows that the normal stress *σ_n_* has the highest ρ of 0.966 in all other parameters, validating the sensitivity analysis results.

### 5.4. Taylor Diagram

**Figure 5:**
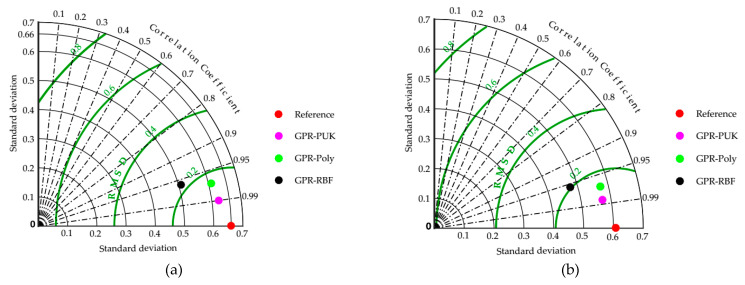
Taylor diagram for (**a**) training dataset (**b**) testing dataset, comparing all the models proposed in the present study.

The Taylor diagram [47] is a simple visual depiction of a model’s performance compared to other models. Three indices are represented in the Taylor diagram: the correlation coefficient, the standard deviation, and the root mean square difference (RMSD). The model outcomes are compared in the Taylor diagram displayed in Figure 5 for a more in-depth examination of the results. The Taylor diagram, which compares the standard deviation (vertical and horizontal axes), correlation coefficient (radial lines), and RMSD, is a valuable tool for illustrating the accuracy of prediction models (green circular lines). The most accurate model, indicated by a pink dot (i.e., GPR-PUK), is introduced as having a similar standard deviation, higher correlation, and reduced RMSD when evaluating real values in the training and testing datasets. Figure 5 shows that the GPR-PUK model is closer to the red dot (actual/reference values) than the other GPR-RBF and GPR-poly models, indicating that this model is accurate.

## 6. Summery and Conclusions

In this research, efforts have been made to create various kernel function-based regression models, i.e., GPR-RBF, GPR-Poly, and GPR-PUK, that may be used to predict the shear strength of RFMs. To train and test the development models, a database from the published literature with different values of influential parameters on RFMs, including *D*_10_, *D*_30_, *D*_60_, *D*_90_, *UCS_min_*, *UCS_max_*, *FM*, *GM*, *γ*, *σ_n_*, and *R*, is considered. The data are split into two categories: training set (80%) and testing set (20%). The output shear strength (τ) of the developed models was evaluated using statistical parameters, including R^2^, *r*, MAE, RMSE, RAE, and RRSE. Furthermore, visual inspection, such as with scatter plots, was also used to assess the effectiveness of the developed models. The applications for the aforementioned models for predicting the shear strength of RFMs were compared and discussed. The following conclusions are made based on the obtained results of this study:

1. GPR-PUK achieved an R-squared (R^2^) of 0.9806, a correlation coefficient (*r*) of 0.9903, a mean absolute error (MAE) of 0.0646 MPa, a root mean square error (RMSE) of 0.0965 MPa, a relative absolute error (RAE) of 13.0776%, and a root relative squared error (RRSE) of 14.6311% in the training phase. In the testing phase, it performed equally well, with R^2^ = 0.9455, *r* = 0.9724, MAE = 0.1048 MPa, RMSE = 0.1443 MPa, RAE = 21.8554%, and RRSE = 23.6865%. The GPR-PUK model was found to be more accurate and stable than the other models. Furthermore, the PUK kernel model had a superior agreement to the observed data based on the scatter plots of actual and predicted values, indicating that it has the potential for wider applications in RFM properties prediction.
2. The results of the sensitivity analysis show that the degree of importance of different input parameters for predicting the shear strength of RFMs is as follows: *σ_n_* > *D*_90_ > *γ* > *R* > *FM* > *UCS_min_*> *D*_60_ > *GM* > *UCS_max_* > *D*_30_ > *C_c_* > *C_u_* > *D*_10_.
3. The developed PUK kernel model makes predictions as accurate as those made by other soft computing techniques. This research also points out that these machine learning techniques can be a potential approach for estimating basic soil parameters, such as the soil permeability coefficient.

GPR-PUK can be used to predict the shear strength of RFMs with high accuracy, according to this study. The sample size is, however, limited. As a result, this study should be extended to include a larger sample size. Furthermore, future studies using other algorithms, such as XGBoost, evolutionary polynomial regression, and gene expression programming, should be utilized to evaluate the algorithms’ effectiveness and gain a comprehensive understanding of the techniques used for predicting the shear strength of RFMs.

## Figures and Tables

**Table 1:** The inputs and output of the present study.

S. No.	*D*_10_ (mm)	*D*_30_ (mm)	*D*_60_ (mm)	*D*_90_ (mm)	*C_C_*	*C_U_*	*GM*	*FM*	*R*	*UCS_min_* (MPa)	*UCS_max_* (MPa)	*γ* (KN/m^3^)	*σ_n_* (MPa)	*τ* (MPa)
1	0.4	2.9	9.7	31	2.17	24.25	3.41	5.57	4	50	100	20.8	1.142	0.93
2	0.44	1.5	6.99	27.5	0.73	15.89	3.82	5.16	4	50	100	18.7	0.159	0.189
3	0.4	3.3	10.3	33.3	2.64	25.75	3.32	5.64	4	50	100	21.8	0.344	0.357
. . .	. . .	. . .	. . .	. . .	. . .	. . .	. . .	. . .	. . .	. . .	. . .	. . .	. . .	. . .
163	0.02	0.94	4	18	11.05	200	4.78	4.19	1	1	5	15.4	0.044	0.025
164	20.7	26.7	32.8	53	1.05	1.58	0.89	8.2	5	100	250	15.3	0.115	0.191
165	0.4	2.3	12.2	44.4	1.08	30.5	3.3	5.69	4	50	100	18.7	0.794	0.577
Min	0.01	0.56	1.2	2.6	0.1	1.36	0.2	3	1	1	5	9.32	0.002	0.005
Max	33.9	42.4	80.1	100	22.27	1040	6	8.8	6	250	400	38.9	4.205	3.921
Mean	4.463	7.86	18.28	39.927	2.404	69.561	2.903	6.142	4.327	73.691	168.455	20.799	0.734	0.662
SD	8.875	10.335	14.42	22.432	3.414	193.628	1.278	1.298	0.957	37.975	87.844	4.861	0.785	0.652

**Table 2:** Statistics of parameters of the training and testing datasets.

Statistical Parameter	Dataset	Input Variable													Output Variable
		*D*_10_ (mm)	*D*_30_ (mm)	*D*_60_ (mm)	*D*_90_ (mm)	*C_C_*	*C_U_*	*GM*	*FM*	*R*	*UCS_min_* (MPa)	*UCS_max_* (MPa)	*γ* (KN/m^3^)	*σ_n_* (MPa)	*τ* (MPa)
Minimum	Training	0.010	0.560	1.200	2.600	0.100	1.360	0.200	3.000	1.000	1.000	5.000	9.320	0.002	0.005
	Testing	0.010	0.560	1.200	2.600	0.100	1.470	0.200	3.000	1.000	1.000	5.000	9.320	0.021	0.024
Maximum	Training	33.900	42.400	80.100	100.000	22.270	1040.000	6.000	8.800	6.000	250.000	400.000	38.900	4.205	3.921
	Testing	33.900	42.400	50.000	99.000	22.270	1040.000	6.000	8.800	5.000	100.000	250.000	38.900	3.223	2.492
Mean	Training	4.867	8.465	19.287	40.386	2.199	53.324	2.788	6.250	4.364	75.045	170.682	20.766	0.729	0.660
	Testing	2.887	5.442	14.252	38.091	3.226	134.510	3.365	5.709	4.182	68.273	159.545	20.932	0.756	0.668
Standard deviation	Training	9.179	10.577	15.135	22.018	3.075	156.064	1.243	1.261	0.910	39.230	88.010	4.605	0.780	0.662
	Testing	7.453	9.050	10.349	24.289	4.492	194.958	1.331	1.374	1.131	32.444	87.967	5.854	0.816	0.619

**Table 3:** Pearson correlation coefficients for variable inputs and the target output.

	*D*_10_ (mm)	*D*_30_ (mm)	*D*_60_ (mm)	*D*_90_ (mm)	*C_c_*	*C_u_*	*GM*	*FM*	*R*	*UCS_min_* (MPa)	*UCS_max_* (MPa)	*γ* (KN/m^3^)	*σ_n_* (MPa)	*τ* (MPa)
*D*_10_ (mm)	1													
*D*_30_ (mm)	0.972	1												
*D*_60_ (mm)	0.652	0.802	1											
*D*_90_ (mm)	0.304	0.451	0.758	1										
*C_c_*	−0.203	−0.229	−0.251	−0.214	1									
*C_u_*	−0.171	−0.207	−0.171	−0.032	0.567	1								
*GM*	−0.784	−0.866	−0.849	−0.686	0.345	0.267	1							
*FM*	0.770	0.846	0.824	0.644	−0.357	−0.273	−0.959	1						
*R*	0.297	0.357	0.457	0.304	−0.607	−0.176	−0.465	0.459	1					
*UCS_min_* (MPa)	0.291	0.411	0.644	0.352	−0.358	−0.169	−0.444	0.428	0.838	1				
*UCS_max_* (MPa)	0.376	0.443	0.536	0.254	−0.363	−0.193	−0.454	0.433	0.864	0.945	1			
*γ* (KN/m^3^)	−0.277	−0.237	−0.079	0.080	0.459	0.189	0.203	−0.305	−0.448	−0.227	−0.292	1		
*σ_n_* (MPa)	−0.249	−0.154	0.146	0.413	−0.164	−0.069	−0.086	0.058	0.098	0.089	−0.034	0.195	1	
*τ* (MPa)	−0.238	−0.130	0.210	0.472	−0.187	−0.080	−0.118	0.092	0.150	0.156	0.030	0.185	0.966	1

**Table 4:** Different regression models’ optimal tuning parameters.

Model	Parameters for Optimal Tuning
RBF kernel	{noise = 0.25, gamma = 0.02}
Poly kernel	{noise = 0.5}
PUK kernel	{noise = 0.3, omega = 0.85, sigma = 0.9}

**Table 5:** Statistical indices and error measures between experimental/actual and predicted shear strength of three different models used in present study for RFMs.

Model	Dataset	R^2^	*r*	MAE (MPa)	RMSE (MPa)	RAE (%)	RRSE (%)
GPR-RBF	Training	0.9224	0.9604	0.1565	0.2219	31.7002	33.6238
	Testing	0.9334	0.9661	0.1395	0.1781	29.0963	29.2377
GPR-Poly	Training	0.9430	0.9711	0.1015	0.1605	20.5572	24.3232
	Testing	0.9411	0.9701	0.1092	0.1508	22.783	24.7641
GPR-PUK	Training	0.9806	0.9903	0.0646	0.0965	13.0776	14.6311
	Testing	0.9455	0.9724	0.1048	0.1443	21.8554	23.6865

**Table 6:** The results of the employed models’ rank analysis.

Model	GPR-RBF		GPR-Poly		GPR-PUK	
Parameter	Training	Testing	Training	Testing	Training	Testing
R^2^	1	1	2	2	3	3
*r*	1	1	2	2	3	3
MAE	1	1	2	2	3	3
RMSE	1	1	2	2	3	3
RAE	1	1	2	2	3	3
RRSE	1	1	2	2	3	3
Rank Score	6	6	12	12	18	18
Total Ranking Score (Training and Testing)	12		24		36	
Total Rank	3		2		1	

## Data Availability

The data that support the findings of this study are available from the corresponding author, upon reasonable request.
